# Impact of chronic SSRI administration on memory parameters, lipid components and membrane properties in C57Bl/6J mice

**DOI:** 10.3389/fphar.2026.1769754

**Published:** 2026-04-17

**Authors:** Michał Szuwarzyński, Anna Kostecka, Helena Domin, Joanna Depciuch, Aleksandra Karska, Magdalena Sowa-Kućma, Patrycja Pańczyszyn-Trzewik, Katarzyna Stachowicz

**Affiliations:** 1 Academic Centre for Materials and Nanotechnology, AGH University of Krakow, Krakow, Poland; 2 Maj Institute of Pharmacology, Polish Academy of Sciences, Krakow, Poland; 3 Institute of Nuclear Physics, Polish Academy of Science, Krakow, Poland; 4 Department of Human Physiology, Faculty of Medicine, University of Rzeszów, Rzeszów, Poland; 5 Centre for Innovative Research in Medical and Natural Sciences, Collegium Medicum, University of Rzeszów, Rzeszów, Poland

**Keywords:** atomic force microscopy, cognition, escitalopram, fluoxetine, lipids, membrane fluidity, modified Barnes maze

## Abstract

**Background:**

Selective serotonin reuptake inhibitors (SSRIs) are well-documented for their impact on memory. Fluoxetine has both positive and negative effects on memory depending on the dose used, the length of administration, and the specific memory context. Conversely, escitalopram has been shown to enhance memory parameters. However, both compounds have the ability to modulate synaptic plasticity and neurogenesis. We selected compounds with these specific mechanisms of action to test our hypothesis.

**Objective:**

We postulate that changes in memory parameters induced by SSRIs may result from changes in lipid composition, which affect the physicochemical properties of cell membranes and, consequently, the functional dynamics of memory.

**Methods:**

Memory tests were conducted using modified Barnes maze (MBM) following chronic SSRI administration to evaluate our hypothesis. Subsequently, brain tissue samples were analyzed using Fourier Transform Infrared Spectroscopy (FT-IR) measurement for lipid changes detection, and atomic force microscopy to measure cell membranes’ elastic modulus.

**Results:**

Chronic treatment with fluoxetine (10 mg/kg *i.p.*) possesses a negative impact on the general learning possibilities of mice and a positive impact on spatial learning in MBM. Influences wiping memory traces and working memory. Escitalopram (2 mg/kg *i.p*.) has a positive impact on memory parameters in MBM. Simultaneously, the prefrontal cortex (pFCx), hippocampus (Hc) and amygdala (Am) tissue revealed under the influence of chronic administration of SSRIs showed differences in lipid composition, which correspond with elastic modulus modifications.

**Conclusion:**

Our findings indicate that chronic administration of SSRIs induces region-specific alterations in lipid composition and membrane elasticity, which parallel changes in memory performance. These results support the hypothesis that SSRI-related modulation of memory may be mediated, at least in part, by lipid-dependent regulation of neuronal membrane properties and synaptic plasticity.

## Graphical Abstract

**Figure:**
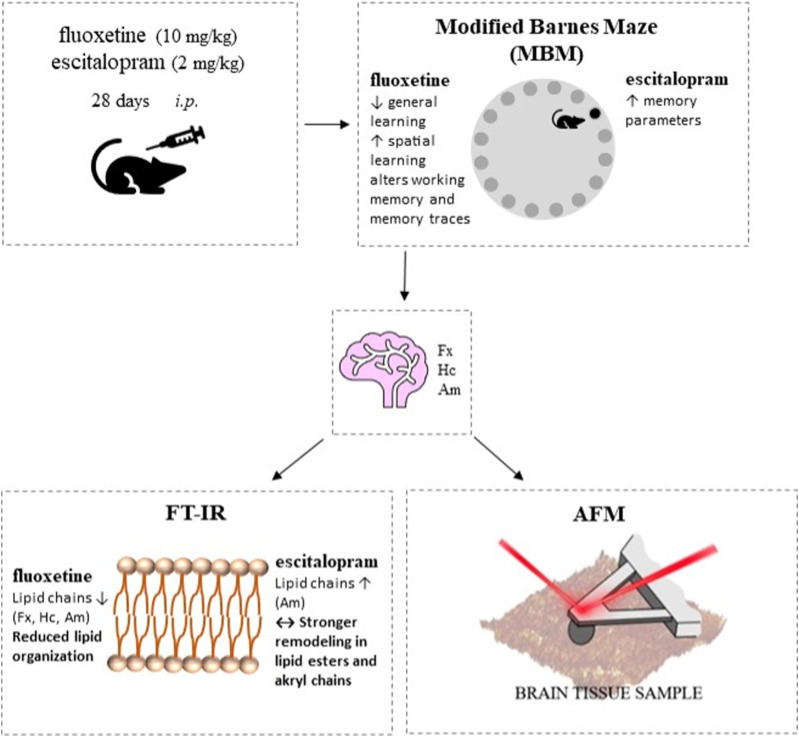

## 1 Introduction

Fluoxetine and escitalopram are selective serotonin reuptake inhibitors (SSRIs) commonly used for the treatment of depression (Adjei et al., 2023). Fluoxetine came out in the early 1990s as one of the first modern antidepressants. In 2003, escitalopram was introduced as part of a newer generation of these drugs (Adjei et al., 2023). SSRIs’ antidepressant action links with the increase of extracellular concentrations of serotonin (5-HT) and amplifies signals sent by 5-HT neurons (Majlessi and Naghdi, 2002; Stachowicz and Sowa-Kućma, 2022). As it states in memory, fluoxetine disrupted memory and, at the same time, improved it, or had no effect on memory parameters (Valluzzi and Chan, 2007). It was test-, dose-, and treatment-dependent (Valluzzi and Chan, 2007; Ma et al., 2017; Meneses and Hong, 1995; Puścian et al., 2021; Majlessi and Naghdi, 2002; Adjei et al., 2023). On the other hand, escitalopram, as an antidepressant of a new generation, possesses beneficial effects on memory parameters (Savaskan et al., 2008; Couto et al., 2012; Farahbakhsh and Radahmadi, 2022).

Learning and memorizing are dynamic sequences of cellular, membrane, and molecular changes. They lead to neuronal communication involving synaptic communication, releasing synaptic vesicles, engaging polyunsaturated fatty acids, and often leading to neurogenesis and plastic changes in the brain (Stachowicz, 2022; Stachowicz, 2023). Fluoxetine and escitalopram, both selective serotonin reuptake inhibitors (SSRIs), have been shown to influence neuroplasticity in the prefrontal cortex (pFCx) and hippocampus (Hc) and to stimulate neurogenesis (Hayley and Littlejohn, 2013; Johansen et al., 2023; Olivas-Cano et al., 2023). Research indicates that these medications increase the expression of brain-derived neurotrophic factor (BDNF), which promotes neuroplasticity processes and neurogenesis in these brain regions (Molteni et al., 2006; Olivas-Cano et al., 2023; Dionisie et al., 2021). Regarding the impact of SSRIs on synaptic vesicle release, available evidence suggests that these drugs may modulate synaptic activity through their interaction with serotonin receptors, indirectly affecting neurotransmitter release (Jung et al., 2014). However, direct studies addressing vesicle release mechanisms influenced by these drugs are limited.

The brain is the second most lipid-rich organ. It mainly contains glycerophospholipids, sphingolipids, and cholesterol, which are components of neuronal membranes. They take part in maintaining the integrity of neuronal membranes, also facilitating synaptogenesis and synaptic plasticity (Sastry, 1985; Fahy et al., 2009; Shamim et al., 2018; Castellanos et al., 2021; Mutlu et al., 2021; He et al., 2025). Lipids modulate membrane fluidity and curvature and organize receptors, all crucial for efficient synaptic signaling (Hering et al., 2003; Cole et al., 2010; Colin et al., 2016; Egawa et al., 2016). Lipid rafts that are microdomains rich in cholesterol and sphingolipids play a central role in organizing synaptic membranes and facilitating local neurotrophic signaling pathways, such as BDNF/TrkB, by ensuring proper receptor localization and activation (Suzuki et al., 2004; Head et al., 2014; Colin et al., 2016; Egawa et al., 2016). These structures impact conformation, trafficking, and signaling of neurotransmitter receptors. This way, they support memory processes and synaptic plasticity (Hering et al., 2003; Head et al., 2014; Colin et al., 2016; Egawa et al., 2016). During aging, neuronal cholesterol levels decline (Martin et al., 2008; Nunes et al., 2022); because cholesterol is critical for synaptic vesicle biogenesis, mobility, presynaptic release, and postsynaptic response, its deficiency may impair synaptic plasticity (Najafinobar et al., 2016; Korinek et al., 2020; Qurban et al., 2025). Besides their structural role, sphingolipids, including sphingosine-1-phosphate, act as bioactive second messengers regulating neuronal survival, differentiation, synaptic function, and hippocampal plasticity (Kanno et al., 2010; Jęśko et al., 2019; Czubowicz et al., 2019). Glycerophospholipids are enzymatically degraded into secondary messengers, such as DAG, which participate in cellular signaling pathways (Farooqui et al., 2010; He et al., 2025). Thus, dysregulations in membrane lipid metabolism may disrupt synaptic function, plasticity, and cognitive processes, learning, and memory processes; a mechanism also observed in neurodegenerative diseases such as Alzheimer’s disease (Farooqui et al., 2010; Castellanos et al., 2021).

The elastic modulus is a critical factor in neurobiology, influencing neuronal development, stem cell differentiation, cellular migration, and the design of neural interfaces (Jiang et al., 2015; Sands et al., 2024). Neurons exhibit varying elastic moduli depending on their type and developmental stage. Atomic force microscopy (AFM) studies have the potential to measure different neurons’ elastic moduli (Spedden et al., 2012; Mustata et al., 2010). These differences are attributed to each neuron type’s cytoskeletal composition and organization. Notably, local stiffening during neurite outgrowth is primarily due to microtubule formation, indicating that changes in elastic modulus are crucial during neuronal growth (Kumarasinghe et al., 2022; Lo et al., 2000; Leipzig and Shoichet, 2009).

As for the effect of fluoxetine and escitalopram on the brain tissue’s elastic modulus (mechanical properties), there is currently a lack of definitive studies examining this aspect. The elastic modulus refers to the mechanical stiffness of a material. In the context of biological tissues, it is an emerging area requiring further investigation, especially about psychotropic drugs. Therefore, further research is needed to better understand their effects on synaptic vesicle release and the mechanical properties of brain tissue.

Taking that all into account, in this study, we conducted a series of experiments utilizing the modified Barnes maze (MBM) to evaluate memory parameters in mice subjected to a 28-day treatment with fluoxetine or escitalopram. Additionally, FT-IR was used to check lipid composition of prefrontal cortex (pFCX), hippocampus (Hc), and amygdala (Amy). AFM was employed to analyze the elastic modulus of brain tissues, specifically the pFCx and Hc. This parameter may play a critical role in influencing the functional dynamics of memory.

## 2 Materials and methods

### 2.1 Materials

The following drugs were used: fluoxetine hydrochloride (Carbosynth limited, United Kingdom); escitalopram (H. Lundbeck A/S, Copenhagen). Gelatin-Coated Slides (75 × 25 mm) (FD NeuroTechnologies, Inc, Columbia, MD, United States).

### 2.2 Animals and housing

The experiments were performed on group-housed male C57BL/6J mice (8–14 weeks old). The animals were kept under guidelines, and food and water were freely available. Experiments were performed during the light period (8:00–18:00). All procedures were conducted according to the National Institutes of Health Animal Care and Use Committee and were approved by the Institute of Pharmacology Ethics Committee, Polish Academy of Sciences in Krakow (Approval Number: 155/2024).

### 2.3 Drug treatment

Fluoxetine (2 mg/kg; 10 mg/kg) and escitalopram (2 mg/kg; 10 mg/kg) were used as aqua solutions. Aqua was used for vehicle group injections. All compounds were injected *i.p.* once daily (before 11:00 a.m.), for 28 days. Intraperitoneal administration was selected to ensure precise dose control and consistent systemic exposure throughout the treatment period. Oral administration better reflects the clinical route in humans. However, intraperitoneal delivery is common in preclinical studies. It reduces variability linked to voluntary intake and absorption differences. The doses used for chronic treatment were selected based on preliminary experiments and available literature data. Lower doses of escitalopram produce stable neurobehavioral effects during prolonged administration. At the same time, it minimizes potential nonspecific behavioral alterations that could interfere with cognitive testing.

### 2.4 Modified Barnes maze test (MBM)

The modified Barnes maze (MBM) test is applied to evaluate the memory of mice (Stachowicz, 2019; Fowler et al., 2013). The Barnes maze consists of a gray circular platform (ϕ122 cm) mounted above the floor (at the height of 113 cm) on a leg with 40 holes and an escape box (17.8 × 5.1 × 10.2 × 7 cm) (Stachowicz, 2019; Fowler et al., 2013). The MBM test includes three stages: training session, retention interval, and test session. The mice were placed individually on a platform and allowed 5-min exploration during training and test sessions. Each animal was tested twice during one session. The following variables were monitored throughout the experiment: latency to find escape hole; the number of errors; strategy; the time spent in a target quadrant that belongs to the escape hole during reversal day #1 - as a measure of memory flexibility in mice; working memory was counted as a difference in time spent in a target quadrant (with an escape hole) between two trials separated by 5 min rest in a home cage; the wiping of memory traces was counted as the number of looking to the old destination hole (plus two adjacent holes, allowing the mouse to make a mistake) during following test days. After each trial, the odor was removed from the platform using a 20% ethanol solution. All stages of the experiment were videotaped.

### 2.5 Tissues collection

24 h after the last experiment, the animals were decapitated, and their brains were removed and stored at −80 °C. Slices were collected according to Paxinos and Franklin, 2001; Paxinos and Franklin, 2001). Cortical slices for nanomechanical characterization were collected in a coronal plane bregma 1.78-1.54; hippocampal slices–bregma (−2.54) – (−2.70); amygdala, using Cryo-cut, and were immediately transferred to a glass slide using a brush dipped in distilled water and frozen at −20 °C until analysis. On the other side brain tissue for lipid composition analysis were collected as follows: The prefrontal cortex (pFCx) was obtained by cutting the anterior part of the forebrain at Bregma 2.20 mm. Olfactory bulbs and the anterior striatum were cut off. Therefore, the tissue taken for analysis contained most of the pFCx. Then, the whole amygdala and HC were taken out from each hemisphere. Before biochemical analysis, the tissues were frozen on dry ice and stored at −80 °C.

### 2.6 FTIR measurement

The Fourier Transform Infrared Spectroscopy (FT-IR) measurements were carried out using a Nicolet IS50 spectrometer equipped with a deuterated triglycine sulfate (DTGS) detector. The attenuated total reflectance (ATR) technique was applied, with the samples placed directly onto a diamond crystal. Prior to each measurement, a background spectrum was recorded. Spectra were acquired in the 4000–400 cm^-1^ range at a resolution of 4 cm^-1^, averaging 32 scans. Data acquisition was performed using OMNIC software (Thermo Fisher Scientific, Waltham, United States), while baseline correction and min–max normalization were carried out in OPUS 7.0. Each sample was measured in triplicate.

### 2.7 Data analyses

To evaluate biochemical variations among the samples, the lipid-specific region of the spectra (2,800–3,000 cm^-1^) was analyzed using Principal Component Analysis (PCA). Although FTIR spectroscopy provides detailed information on molecular vibrations of proteins, lipids, carbohydrates, and nucleic acids, overlapping absorption bands often hinder the identification of subtle differences by visual inspection alone. PCA was therefore applied as a dimensionality reduction method, enabling the extraction of the major sources of variance within the spectral dataset. By converting high-dimensional spectral data into a smaller number of orthogonal components, PCA emphasizes the features that most strongly differentiate the groups. This approach facilitated the visualization of clustering patterns and assessment of whether experimental conditions produced distinct biochemical profiles. Statistical analyses were conducted using Past 4.0 software.

### 2.8 Quantitative nanomechanics (QNM) of murine brain tissues

Atomic Force Microscope (AFM) images were obtained with Dimension Icon XR atomic force microscope (Bruker, Santa Barbara, CA, United States) working in the water in the PeakForce Quantitative Nanomechanical Mapping (PF QNM) mode (Hu et al., 2020; Hinz et al., 2024; Karabrasz et al., 2020). Soft silicon calibrated probes (Bruker) with the nominal spring constant of 0.0014 N/m, resonance frequency of 3.32 kHz, spherical colloidal tip with 10 µm diameter were used for the measurements. The purchased measuring probes were previously calibrated by the manufacturer, which significantly reduced the procedure itself before QNM measurements. The value of the spring constant k given by the manufacturer, along with other parameters of the probe geometry were entered into the program (Nanoscope 9.7, Bruker).The type of AFM probe was selected based on the expected material properties. The probes with the above-mentioned parameters are dedicated for soft and biological samples and are suitable for measuring soft tissue (murine brain) whose elastic modulus range was expected to be in the range of kPa. All samples were prepared by cutting brain tissues on a microtome and placing it on a microscope slide. The prepared samples were kept frozen until the measurement, and they were thawed immediately before the measurement by applying a drop of water in which the measurement was made. Before the measurement, the system was stabilized for 30 min. The measurement parameters for each series were the same and were selected through optimization to enable collecting information on the elasticity modulus while not deforming or destroying the soft sample: PF setpoint of 500 pN, PF amplitude of 100 nm, PF lift of 200 nm. The data were collected in 8 areas with 8 measuring points each on two different groups of samples (128 points in total), were processed using NanoScope Analysis 1.9 (Bruker) and OriginPro 24b (OriginLab) software.

### 2.9 Statistical analysis

The results were presented as the means ± S.E.M. A repeated measure ANOVA using day as a repeated measure was adopted for MBM data calculation. Histogram of frequency distribution was used for analysis of strategy in the MBM test. One-way (followed by the Dunnett’s test) analysis of variance (ANOVA) was used for statistical analysis of selected MBM studies. GraphPad Prism software, ver. 10 (San Diego, CA, United States) was used for one-way ANOVA, and Statistica 13.0 for Windows was used for repeated measure calculation. p < 0.05 was considered significant.

## 3 Results

### 3.1 The effects of chronic treatment with SSRIs (fluoxetine or escitalopram) on behavior of C57Bl/6J mice in MBM

#### 3.1.1 The effects of chronic treatment with SSRIs (fluoxetine or escitalopram) on learning performance of C57Bl/6J mice in MBM

**FIGURE 1:**
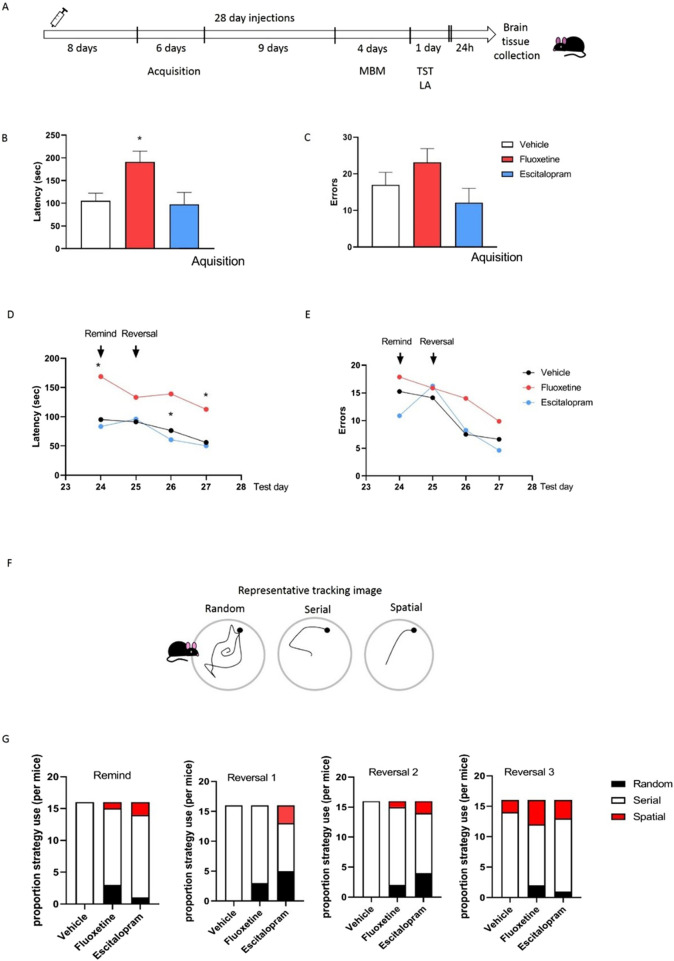
The effect the effects of the treatment for 28 days with fluoxetine (10 mg/kg), or escitalopram (2 mg/kg) on memory performance of C57Bl/6J mice in MBM. **(A)** Presents a schematic diagram of injections and experimental schedule. **(B)** Acquisition experiments–latency to find an escape hole. **(C)** Acquisition experiments–the number of errors; latency to find escape box. **(D)** Latency to find an escape hole. **(E)** The number of errors. **(F)** Representative tracking images. **(G)** Proportion of strategy use. Values are expressed as the means ± S.E.M., and were evaluated by one-way **(B,C)** and repeated measure ANOVA **(D,E)**, and histogram of frequency distribution **(G)**, (n = 8). Asterisk on Figure 1D represents Post Hoc Newman-Keuls test.

Treatment of mice with fluoxetine or escitalopram for 28 days (doses selected in preliminary studies see Supplementary Figure S1 and Supplementary Table S1; Beaver et al., 2022) produced different effects on the learning ability of mice in the MBM (Figure 1). Fluoxetine in a dose of 10 mg/kg impaired the learning process already at the acquisition stage, as the last day of acquisition shows [F(2,21) = 5.221; P = 0.014] (Figures 1B,C). Mice treated with fluoxetine needed significantly more time to find safe refuge (Figure 1B), and made more mistakes (Figure 1C). Similar changes were found during remind phase and reversal (Figures 1D,E). A repeated measure ANOVA showed a significant effect of the treatment on latency to find the escape box in the MBM [F(2,21) = 3.59; P = 0.04] demonstrating that mice in fluoxetine group had impaired learning, however all groups made progress while repeated measure ANOVA demonstrated significant effect of the day of the test [F(3,63) = 5.70; P = 0.001] (Figure 1D). As it states to number of errors mice made during MBM, repeated measure ANOVA found no effect of the treatment on the number of errors made in MBM test [F(2,21) = 1.49; P = 0.24], and a significant effect of the day [F(3,63) = 13.23; P = 0.0001], demonstrating that all mice made a significant progress during the test (Figure 1E). Representative tracking image of strategy mice choose is presented in (Figure 1F). It is interesting, that mice treated chronically with fluoxetine (10 mg/kg) use the most spatial strategy during the last day of the test (about 25%) when comparing to the vehicle mice (about 13%) or escitalopram mice (about 19%) (Figure 1G).

#### 3.1.2 The effects of chronic treatment with SSRIs (fluoxetine or escitalopram) on wiping memory traces, working memory and memory flexibility of C57Bl/6J mice in MBM

**FIGURE 2:**
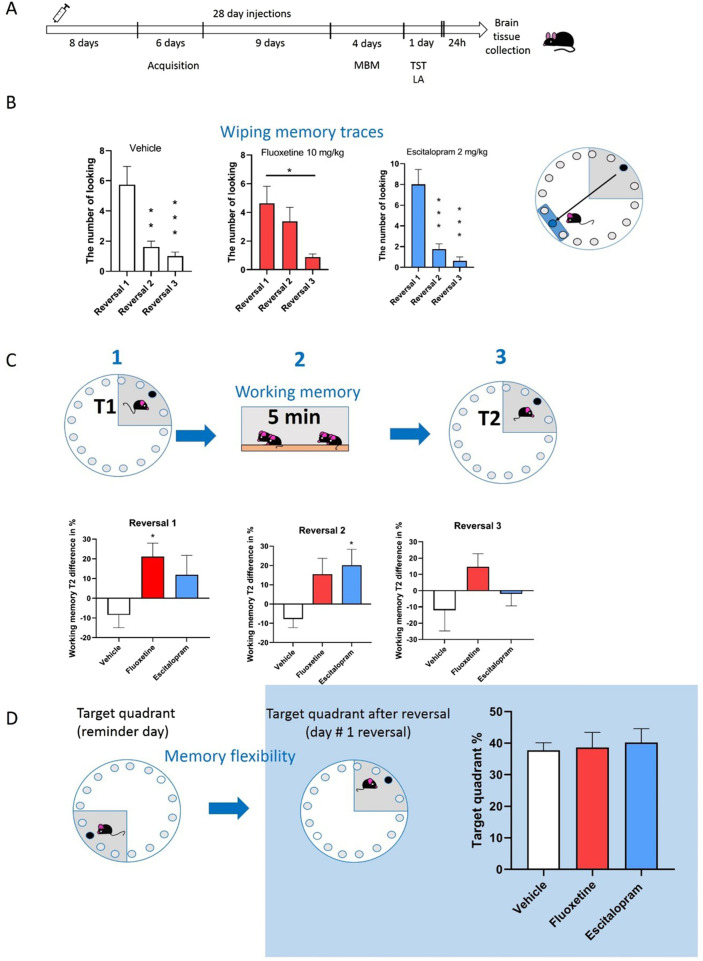
The effects of the treatment for 28 days with fluoxetine (10 mg/kg), or escitalopram (2 mg/kg) on selected memory parameters in MBM. **(A)** Schematic diagram of injections and experimental schedule using MBM. **(B)** The effects of the treatment for 28 days with fluoxetine (10 mg/kg), or escitalopram (2 mg/kg) on wiping memory traces in MBM. **(C)** The effects of the treatment for 28 days with fluoxetine (10 mg/kg), or Escitalopram (2 mg/kg) on working memory in MBM. **(D)** The effects of the treatment for 28 days with fluoxetine (10 mg/kg), or escitalopram (2 mg/kg) on memory flexibility in MBM. One-way ANOVA, followed by Dunnet, n = 8. Values are expressed as the means ± S.E.M., *P < 0.05, **P < 0.01, ***P < 0.001 vs. vehicle group. Asterisk on Figure 1 represents Post Hoc Newman-Keuls test.

Wiping memory traces: Vehicle-treated mice for 28 days have (Figure 2A) shown the successive forgetting of the old location of the safe hole on the labyrinth with each passing day [F(2,21) = 11.99; P = 0.0003] (Figure 2B). Similar observation was find in escitalopram group [F(2,21) = 18.76; P < 0.0001]. However, mice treated with fluoxetine for 28 days needed more time to start forgetting the old location of the safe hole [F(2,21) = 4.483; P = 0.024].

Working memory of mice functioned differently after treatment with vehicle, fluoxetine or escitalopram, another variable modulating working memory was days after table reversal (Figure 2C). During reversal #1 day mice treated with fluoxetine showed significant difference in time spend in target quadrant during second exposition (after 5 min in a home cage) [F(2,21) = 3.698; P = 0.042]. Oppositely, during #2 reversal day significant differences were induced by escitalopram [F(2,21) = 4.251; P = 0.028]. During #3 days after reversal, mice showed less engagement of working memory to find shelter [F(2,21) = 1.901; P = 0.174]. The difference in time spent in the target quadrant between the two trials was used as a working memory. These trials were separated by a short interval. Negative values indicate that the animal spent less time in the target quadrant (during the second trial than during the first). This pattern reflects variability in exploratory behavior, not an absence of intact working memory.

All mice, irrespective of the type of drug administered, showed a similar memory flexibility [F(2,21) = 0.0897; P = 0.915] (Figure 2D).

### 3.2 The effects of chronic treatment with SSRIs (fluoxetine or escitalopram) on lipid composition of prefrontal cortex, hippocampus and amygdala of C57Bl/6J mice

**FIGURE 3:**
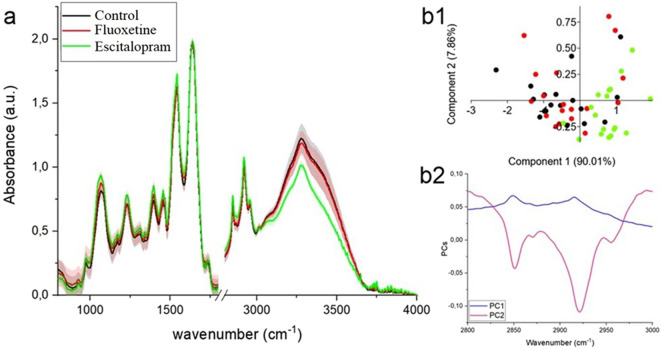
**(a)** FRIR spectra ±SD of amygdala collected from control group (black color), fluoxetine group (red color) and escitalopram group (green color); PCA analysis **(b1)** with loading plots **(b2)** for FTIR lipids region (2,800–3,000 cm^-1^).

**FIGURE 4:**
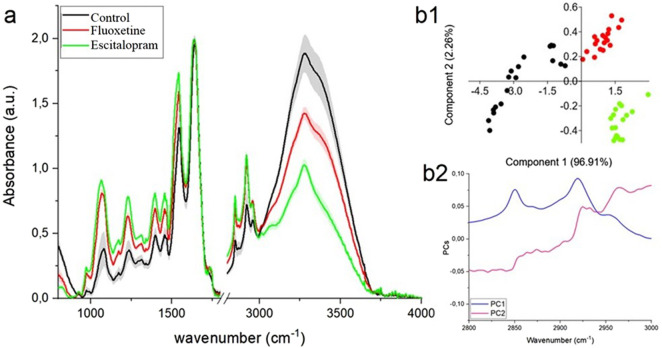
**(a)** FRIR spectra ±SD of hippocampus collected from control group (black color), fluoxetine group (red color) and escitalopram group (green color); PCA analysis **(b1)** with loading plots **(b2)** for FTIR lipids region (2,800–3,000 cm-1).

**FIGURE 5:**
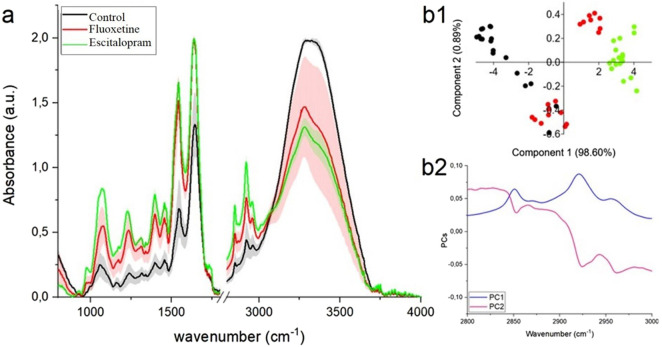
**(a)** FRIR spectra ±SD of prefrontal cortex collected from control group (black color), fluoxetine group (red color) and escitalopram group (green color); PCA analysis **(b1)** with loading plots **(b2)** for FTIR lipids region (2,800–3,000 cm^-1^).

In the FTIR spectra obtained from all analyzed brain regions: amygdala, hippocampus, and prefrontal cortex (Figures 3–5, respectively) characteristic vibrational modes associated with carbohydrates, phospholipids, proteins, and lipids can be clearly observed. The band around ∼970 cm^-1^ corresponds to C–H out-of-plane deformations of unsaturated lipids. At approximately ∼1,080 cm^-1^, a strong signal arises from the symmetric stretching of phosphate groups (PO_2_^−^) in phospholipids, while the absorption near ∼1,160 cm^-1^ is related to C–O–C and C–O stretching vibrations typical of carbohydrates. The asymmetric PO_2_^−^ stretching of phospholipids is visible around ∼1,240 cm^-1^. Protein-related contributions appear in the Amide III region at ∼1,320 cm^-1^, followed by a band at ∼1,400 cm^-1^ attributed either to COO^−^ symmetric stretching or CH_2_/CH_3_ bending modes arising from both proteins and lipids. The signal at ∼1,460 cm^-1^ is also associated with CH_2_ scissoring and CH_3_ bending vibrations characteristic of these biomolecules. Further protein features include the Amide II band at ∼1,540 cm^-1^ (N–H bending coupled with C–N stretching) and the prominent Amide I band at ∼1,650 cm^-1^, arising from C=O stretching of peptide bonds. Lipid-specific ester C=O stretching is evident at ∼1740 cm^-1^. In the higher wavenumber region, several strong absorptions correspond to lipid alkyl chains: the CH_2_ symmetric and asymmetric stretching modes appear at ∼2,850 cm^-1^ and ∼2,925 cm^-1^, respectively, while the CH_3_ asymmetric stretching vibration is observed near ∼2,965 cm^-1^. Finally, the broad absorption around ∼3,270 cm^-1^ is characteristic of N–H stretching, commonly referred to as the Amide A band.

#### 3.2.1 Amygdala

When comparing the spectra, both fluoxetine group and escitalopram group exhibit noticeable differences relative to the control. In particular, the absorbance in the lipid-associated CH_2_ and CH_3_ stretching region (2,800–3,000 cm^-1^) is reduced in fluoxetine group, indicating a lower relative content of lipid chains. Escitalopram group, in contrast, shows slightly higher intensities in this region compared to both the control and fluoxetine group, suggesting an increased lipid contribution, Figure 3a. The PCA score plot (Figure 3b1) clearly separates the groups, with PC1 accounting for over 90% of the variance. The loading plot (Figure 3b2) highlights that the major discriminating features correspond to the CH_2_ symmetric (∼2,853 cm^-1^), CH_2_ asymmetric (∼2,924 cm^-1^), and CH_3_ asymmetric (∼2,964 cm^-1^) stretching vibrations. This confirms that the main biochemical differences between the groups are related to alterations in lipid composition and chain structure within the amygdala.

From Table 1 it was noticed that in fluoxetine group and escitalopram group, in comparison with control group, shift of peaks originated from PO_2_^−^ asymmetric stretching vibrations from phospholipids, CH_2_ scissoring/CH_3_ bending functional group from lipids and proteins, amide I bands were visible. Additional, in escitalopram group, shift of peak corresponding from C–O–C stretching/C–O stretching from carbohydrates was visible in comparison with control one. These shift means structural changes in chemical compounds building by these functional groups.

**TABLE 1:** Position of peaks visible in Figure 3, where “*” means significant shift vs. Control (higher than 4 cm^-1^).

Control	Fluoxetine group	Escitalopram group	Band assigned
979	981	973	C–H out-of-plane/C–H deformation of = CH (unsaturated lipids)
1,053	1,060 *	1,073 *	PO_2_^−^ symmetric stretching (phospholipids)
1,176	1,173	1,165 *	C–O–C stretching/C–O stretching (carbohydrates)
1,244	1,242	1,238	Asymmetric PO_2_^−^ stretching (phospholipids)
1,318	1,320	1,320	Amide III
1,403	1,401	1,399	COO^−^ symmetric stretching or CH_3_/CH_2_ bending (lipids and proteins)
1,442	1,453 *	1,457 *	CH_2_ scissoring/CH_3_ bending (lipids and proteins)
1,544	1,542	1,544	Amide II band (N–H bending + C–N stretching)
1,640	1,646 *	1,647 *	Amide I band (C=O stretching of peptide bond)
1744	1740	1744	C=O stretching of ester groups (lipids)
2,853	2,854	2,853	CH_2_ symmetric stretching (lipids)
2,926	2,925	2,924	CH_2_ asymmetric stretching (lipids)
2,965	2,966	2,964	CH_3_ asymmetric stretching (lipids)
3,268	3,267	3,268	N–H stretching (amide A)

#### 3.2.2 Hippocampus

In the hippocampal spectra, both experimental groups display lower absorbance compared to the control across the lipid-associated CH_2_ and CH_3_ stretching region. Fluoxetine group shows a moderate decrease in band intensities, while escitalopram group exhibits a more pronounced reduction, particularly in the CH_2_ symmetric (∼2,853 cm^-1^) and CH_2_ asymmetric (∼2,924 cm^-1^) stretching modes, Figure 4a. This suggests a progressive decline in lipid chain content and structural order relative to the control. The PCA score plot (Figure 4b1) demonstrates a clear separation of all three groups, with PC1 accounting for nearly all the variance (96.91%). The loading plot (Figure 4b2) indicates that the discriminating variables are primarily linked to lipid methylene and methyl stretching vibrations, confirming that alterations in lipid composition represent the major source of biochemical differences in the hippocampus between the groups.

From Table 2 it can be observed that both fluoxetine group and escitalopram group exhibited several peak shifts when compared with the control. In particular, displacements were noted in the bands originating from PO_2_^−^ symmetric and asymmetric stretching vibrations of phospholipids, CH_2_ scissoring/CH_3_ bending associated with lipids and proteins, and the Amide II region. In escitalopram group, additional shifts appeared in bands assigned to C–H out-of-plane deformation of unsaturated lipids, C–O–C/C–O stretching of carbohydrates, and COO^−^ symmetric stretching. Moreover, a pronounced shift in the ester C=O stretching vibration (∼1740–1748 cm^-1^) and in the CH_2_ and CH_3_ asymmetric stretching modes (∼2,923 and 2,960 cm^-1^) was detected in escitalopram group compared to the control, indicating changes in lipid ester groups and alkyl chain dynamics. A slight shift of the N–H stretching band (Amide A) was also evident in fluoxetine group. These modifications suggest structural alterations in phospholipids, proteins, lipids, and carbohydrates, reflecting biochemical remodeling in the hippocampal tissue under experimental conditions.

**TABLE 2:** Position of peaks visible in Figure 4, where “*” means significant shift vs. Control (higher than 4 cm^-1^).

Control	Fluoxetine group	Escitalopram group	Band assigned
986	990	975 *	C–H out-of-plane/C–H deformation of = CH (unsaturated lipids)
1,085	1,083	1,071 *	PO_2_^−^ symmetric stretching (phospholipids)
1,188	1,186	1,182 *	C–O–C stretching/C–O stretching (carbohydrates)
1,238	1,235	1,219 *	Asymmetric PO_2_^−^ stretching (phospholipids)
1,319	1,321	1,320	Amide III
1,392	1,405 *	1,401 *	COO^−^ symmetric stretching or CH_3_/CH_2_ bending (lipids and proteins)
1,465	1,467	1,451 *	CH_2_ scissoring/CH_3_ bending (lipids and proteins)
1,550	1,543 *	1,537 *	Amide II band (N–H bending + C–N stretching)
1,647	1,645	1,648	Amide I band (C=O stretching of peptide bond)
1740	1743	1748 *	C=O stretching of ester groups (lipids)
2,857	2,853	2,853	CH_2_ symmetric stretching (lipids)
2,929	2,925	2,923 *	CH_2_ asymmetric stretching (lipids)
2,967	2,965	2,960 *	CH_3_ asymmetric stretching (lipids)
3,293	3,298 *	3,291	N–H stretching (amide A)

#### 3.2.3 Prefrontal cortex

In the prefrontal cortex, both experimental groups show a marked decrease in absorbance compared to the control, particularly within the CH_2_ symmetric (∼2,853 cm^-1^), CH_2_ asymmetric (∼2,924 cm^-1^), and CH_3_ asymmetric (∼2,964 cm^-1^) stretching vibrations characteristic of lipids. Fluoxetine group presents a moderate reduction, whereas escitalopram group exhibits an even stronger decrease, indicating a more pronounced decline in lipid content and structural integrity, Figure 5a. The PCA score plot (Figure 5b1) demonstrates clear segregation of the three groups, with PC1 explaining nearly all of the variance (98.60%). The loading plot (Figure 5b2) further confirms that the differences are primarily driven by methylene and methyl stretching modes, highlighting that lipid alterations are the major biochemical factor distinguishing the prefrontal cortex spectra across the groups.

From Table 3 it can be seen that both experimental groups showed distinct peak shifts compared with the control in the prefrontal cortex spectra. In fluoxetine group, significant displacements occurred in the bands assigned to PO_2_^−^ symmetric and asymmetric stretching of phospholipids, C–O–C/C–O stretching of carbohydrates, CH_2_ scissoring/CH_3_ bending modes of lipids and proteins, as well as in the COO^−^ stretching region. Escitalopram group revealed even more pronounced changes, including shifts in the Amide III and Amide II bands, together with alterations in the CH_2_ asymmetric and CH_3_ asymmetric stretching vibrations, reflecting modifications in protein secondary structure and lipid chain organization. Furthermore, fluoxetine group displayed a notable shift in the N–H stretching vibration (Amide A), while escitalopram group maintained a value close to the control. Altogether, these shifts indicate substantial structural rearrangements in carbohydrates, phospholipids, lipids, and proteins of the prefrontal cortex, with escitalopram group showing the strongest spectral alterations relative to the control.

**TABLE 3:** Position of peaks visible in Figure 5, where “*” means significant shift vs. Control (higher than 4 cm^-1^).

Control	Fluoxetine group	Escitalopram group	Band assigned
​	978 *	993 *	C–H out-of-plane/C–H deformation of = CH (unsaturated lipids)
1,066	1,086 *	1,068	PO_2_^−^ symmetric stretching (phospholipids)
1,170	1,186 *	1,161 *	C–O–C stretching/C–O stretching (carbohydrates)
1,248	1,241 *	1,239 *	Asymmetric PO_2_^−^ stretching (phospholipids)
1,317	1,317	1,323 *	Amide III
1,407	1,402 *	1,391 *	COO^−^ symmetric stretching or CH_3_/CH_2_ bending (lipids and proteins)
1,453	1,465 *	1,467 *	CH_2_ scissoring/CH_3_ bending (lipids and proteins)
1,541	1,545	1,546 *	Amide II band (N–H bending + C–N stretching)
1,650	1,644 *	1,649	Amide I band (C=O stretching of peptide bond)
1747	1746	1746	C=O stretching of ester groups (lipids)
2,857	2,856	2,857	CH_2_ symmetric stretching (lipids)
2,931	2,919 *	2,925 *	CH_2_ asymmetric stretching (lipids)
2,970	2,965 *	2,966	CH_3_ asymmetric stretching (lipids)
3,296	3,262 *	3,297	N–H stretching (amide A)

### 3.3 Nanomechanical characteristics of murine cortex and hippocampus after chronic treatment with SSRIs (fluoxetine or escitalopram)

**FIGURE 6:**
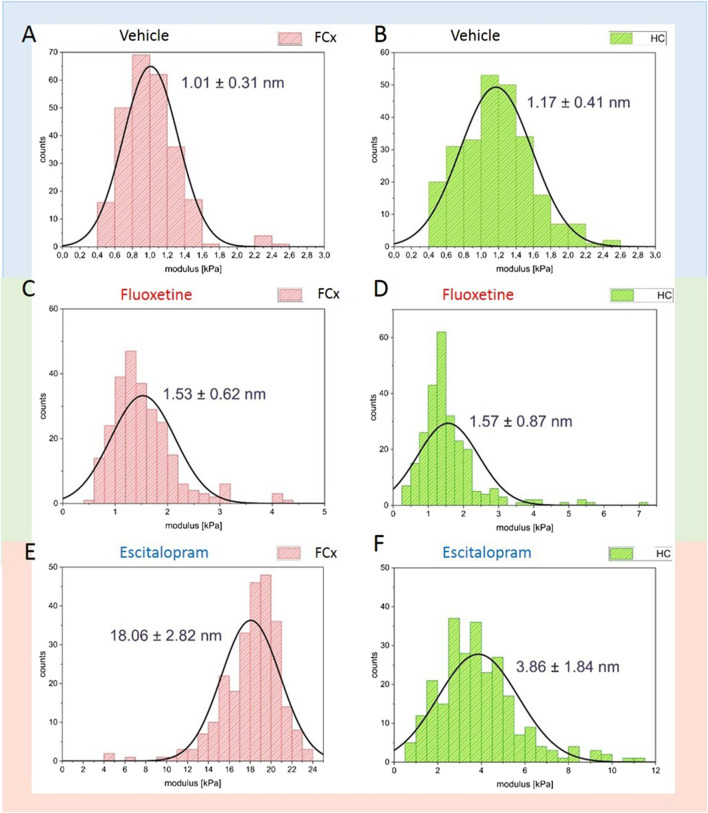
Elastic modulus of murine prefrontal cortex (pFCx–red histogram) and hippocampus (HP–green histogram): **(A,B)** bare tissue, **(C,D)** fluoxetine treatment and **(E,F)** escitalopram treatment. Chronic treatment.

To characterize nanomechanical properties of murine tissue we used quantitative nanomechanical measurements (QNM) (Exton et al., 2023) using atomic force microscopy in the water. Two different regions of murine brain was studied: prefrontal cortex (pFCx) and hippocampus (HC). Both sample types were investigated after chronic treatment with fluoxetine and escitalopram, and the results were compared with control bare vehicle samples. An appropriate calibrated measuring colloidal probe with a diameter of 10 µm was intended for testing soft matter in such a way as to obtain information about the mechanical properties of all tested samples. Due to the probe parameters (low tip radius, spherical shape, low cantilever spring constant), the gathered images were characterized by poor resolution on the plane, but our studies were focused on gathering information about the elastic modulus (DMT modulus) calculated from force-distance curves. The vehicle sample tissues behaved like a model tissue, with the elastic modulus of 1.01 ± 0.31 kPa for pFCx and 1.17 ± 0.41 kPa for HC (Figures 6A,B). The modulus value for samples after chronic fluoxetine treatment increased only slightly, which shows the small effect of this substance on the tissue structure. Elastic modulus values were 1.53 ± 0.62 kPa for pFCx and 1.57 ± 0.87 kPa for HC (Figures 6C,D). The largest change was observed for pFCx treated with escitalopram, for which the modulus value increased to 18.06 ± 2.82 kPa. For HC samples treated with this substance (Figures 6D,F), an increase in modulus to 3.86 ± 1.84 kPa was observed, however, although this value is more than twice that of the reference bare tissue, it is significantly lower than the value for escitalopram for pFCx.

## 4 Discussion

Here, we posited that alterations in memory parameters induced by selective serotonin reuptake inhibitors (SSRIs) may stem from modifications in a physicochemical property of brain cell membranes, specifically the elastic modulus. To examine this hypothesis, we selected two compounds with contrasting effects on rodent behaviour within the studied context (Valluzzi and Chan, 2007; Ma et al., 2017; Meneses and Hong, 1995; Puścian et al., 2021; Majlessi and Naghdi, 2002; Adjei et al., 2023; Savaskan et al., 2008; Couto et al., 2012; Farahbakhsh and Radahmadi, 2022). Subsequently, we obtained brain tissue from mice subjected to MBM learning and chronically treated with fluoxetine or escitalopram, enabling final lipid composition and elasticity modulus testing using atomic force microscopy (AFM).

### 4.1 Impact of SSRIs on memory parameters in MBM

In our study, chronic administration of fluoxetine (10 mg/kg) significantly impeded the learning performance of mice in the MBM task, in contrast to escitalopram (2 mg/kg). These findings constitute the first account of mouse behaviour in the MBM paradigm under the influence of the specific SSRIs examined. Fluoxetine, instead of escitalopram, exhibited a deleterious effect on learning during the acquisition phase, which persisted throughout the testing period. Conversely, fluoxetine demonstrated a more pronounced enhancement of spatial learning; however, this effect only emerged on the final day of testing, suggesting a requirement for extended time to manifest. In contrast, escitalopram exhibited an earlier but comparatively weaker impact on spatial learning, observable from the initial day of testing.

Mice treated with fluoxetine appeared to place greater demands on their working memory, with the benefits to spatial learning and memory utilization emerging notably later. This delay may indicate an intellectual effort not evident in the escitalopram-treated cohort. Similar observations have been reported by Valluzzi and Chan (2007), who proposed that fluoxetine modulates hippocampal-dependent and hippocampal-independent learning abilities in distinct ways. They suggested that fluoxetine’s effects are brain-structure-specific (Valluzzi and Chan, 2007). Such mechanisms may account for the diverse and often contradictory findings concerning cognitive alterations associated with fluoxetine. Reports in the literature highlight a spectrum of effects, including impaired spatial learning (Majlessi and Naghdi, 2002), deficits in serial pattern learning during the serial multiple-choice task (Sharp et al., 2019), and diminished motivation and reward learning (Puścian et al., 2021).

Nevertheless, this explanation does not fully elucidate discrepancies observed in studies assessing identical memory contexts that presumably engage the same neural substrates - for example, fluoxetine-induced enhancements in spatial memory reported by Ma et al. (2017). It is plausible that multiple interacting factors, including the specific brain structures involved, the memory context under investigation, the dosage and regimen of fluoxetine administration, and the species employed in the study, collectively shape the drug’s impact on memory parameters (Ma et al., 2017).

Significantly, human studies on fluoxetine have also been conducted (Ampuero et al., 2024). Nevertheless, similar inconsistencies emerge, influenced by variables such as gender, treatment duration, age, underlying disease pathology, and the nuances of cognitive assessments (Ampuero et al., 2024).

In contrast, escitalopram is not subject to such controversies regarding its impact on memory parameters. Its positive influence on memory has been well-documented (Farahbakhsh and Radahmadi, 2022; Couto et al., 2012; Savaskan et al., 2008), as corroborated by our findings. In this study, we demonstrate that the compounds under investigation exhibit divergent effects on memory and learning in mice. These results align with our hypothesis, which aims to examine the physicochemical properties of brain tissue following exposure to compounds with opposing effects on memory, thereby enabling us to elucidate the underlying differences, should our hypothesis prove accurate.

### 4.2 Impact of SSRIs on lipid composition of brain tissue

Our FT-IR analysis clearly demonstrates that chronic administration of fluoxetine and escitalopram induces region-specific remodeling of brain lipid composition. A decrease in the intensity of CH_2_/CH_3_ stretching vibrations (2,800–3,000 cm^-1^) in the amygdala and hippocampus of fluoxetine-treated mice indicates a reduction in lipid chain content, whereas escitalopram produced an opposite effect in the amygdala, suggesting an increase in lipid contribution. Similar decreases in hippocampal and prefrontal cortex spectra were observed in both experimental groups, with escitalopram producing stronger band shifts, particularly in the ester C=O and CH_2_/CH_3_ modes, pointing to changes in lipid ester groups and alkyl chain organization. These spectral changes may be compatible with modifications in membrane phospholipid, sphingolipid, and cholesterol composition or packing, which can influence membrane fluidity and receptor signaling and thereby potentially affect synaptic plasticity and memory-related processes (Egawa et al., 2016; Egawa et al., 2018; Stuchtey et al., 2022; Vanicek et al., 2022; McNamara et al., 2010). Membrane lipid rafts are enriched in cholesterol, and are also a building blocks of synapses, influencing synaptic plasticity (Egawa et al., 2016). Decrees or decomposition of cholesterol with age were shown connected with cognitive disturbances (Egawa et al., 2016), and this indicates that the path we have taken seems to be the right one.

Escitalopram presented a different profile, producing pronounced shifts in phosphate and amide bands while exerting a weaker effect on CH_2_/CH_3_ intensities. This pattern may reflect a more complex reorganization of phospholipid head groups and protein–lipid interactions, rather than a simple reduction of lipid chains. Such differences may underlie the more favorable cognitive effects of escitalopram observed in behavioral testing and reported by other groups. It was documented that prolonged escitalopram treatment may be connected with neuroplastic changes in the left dorsolateral prefrontal cortex (Vanicek et al., 2022). In these patients that developed sufficient escitalopram blood levels of the drug–increased gray matter density was detected (Vanicek et al., 2022). Based on current evidence, escitalopram treatment is associated with measurable alterations in brain lipid composition, including hippocampal lipid remodeling and changes in specific phospholipid classes. Studies in animal models and cell systems further demonstrate that escitalopram accumulates in membrane lipid rafts and reorganizes membrane microdomains, thereby influencing signaling protein localization. These findings support the hypothesis that escitalopram exerts part of its antidepressant action through modulation of neuronal membrane lipid organization and dynamics (MahmoudianDehkordi et al., 2021; Bussmann et al., 2024; Zhang and Rasenick, 2010; Erb et al., 2016). Studies on lipid-raft accumulation indicate that escitalopram partitions into cholesterol- and sphingolipid-rich membrane domains, a process that may subtly alter bilayer fluidity, thickness, and curvature, although the precise structural consequences remain to be fully established (Erb et al., 2016).

Together with the parallel modifications in tissue elastic modulus detected by AFM, our data support the hypothesis that SSRI-induced modulation of memory is at least partly mediated by lipid-dependent regulation of neuronal membrane properties and synaptic plasticity.

### 4.3 Impact of SSRIs on elastic modulus detected by atomic force microscope (AFM)

Brain murine tissue samples from pFCx and HC exposed to fluoxetine escitalopram showed stiffening of membranes. The difference in value depends on the type of substance administered and the location in the brain tissue. The values obtained for the reference sample for pFCx and HC assumed a mean value of 1.01 ± 0.31 kPa for pFCx and 1.17 ± 0.41 kPa for HC, which is consistent with what can be found in the literature (MacManus et al., 2017; Antonovaite et al., 2021; Eggeling et al., 2009). Literature values range from 0.4 to 12 kPa, and the differences result from animal-related factors such as gender, age, animal husbandry conditions, as well as the measurement). conditions themselves–sample slice preparation, cantilever properties (Antonovaite et al., 2018). In our study, the effect of fluoxetine was relatively small, increasing the elastic modulus to 1.53 ± 0.62 kPa for pFCx and 1.57 ± 0.87 Pa for HC. It is clear that this substance does not interact with the phospholipid membrane in a persistent manner, even after chronic treatment for 28 days. Nor can we identify a preferred area in the animal’s brain that might interact more clearly with fluoxetine. The situation is completely different after chronic 28-day treatment of escitalopram, whose effect is visible not only in the increase in the elastic modulus of the tissue but, above all, in the different affinities observed depending on the brain fragment type. The value for pFCx was 18.06 ± 2.82 kPa, which is an order of magnitude higher than that of pure pFCx tissue. The modulus of value 3.86 ± 1.84 kPa obtained for HC samples is more than three times more as for the reference HC sample, but it is significantly lower than for the pFCx fragment of the same brain. The results obtained from PF-QNM measurements are consistent with previous results from the modification of murine tissue with NS398, a selective COX-2 inhibitor (Lachowicz et al., 2026; in review). In that and in this cases, a change in the plasticity of the sample was observed, which allows us to clearly state that the influence of the administered substances has an impact on the structure and elasticity of the brain tissue.

### 4.4 Limitations

One limitation of the present study is that the experiments were performed in healthy mice. Future experiments should use animal models exhibiting depression-like phenotypes. The primary aim of this work was to investigate the basal effects of chronic SSRI administration on neuronal membrane lipid composition and nanomechanical properties. Our experiments try to explore their potential relationship with memory parameters. For this reason, healthy animals were used to avoid confounding neurochemical and metabolic alterations from chronic stress paradigms. Future studies should investigate whether similar lipid remodeling and nanomechanical changes occur in models of depression, such as chronic stress or social isolation paradigms. Doing so would further strengthen the translational relevance of these findings.

Another limitation is that all animals undergo behavioral testing before tissue collection. All experimental groups were exposed to the same behavioral protocol, which minimizes potential confounding effects of learning-induced plasticity. Furthermore, the brain tissue was collected 24 h after the last experiment. However, it cannot be fully excluded that cognitive stimulation itself may contribute to subtle biochemical changes in neuronal membranes. Future studies that include additional control groups receiving drug treatment without behavioral testing would help disentangle pharmacological and learning-related effects on lipid composition.

Another limitation of this study is that only male mice were used. The decision to use males was made first to reduce variability from hormonal cycle-related fluctuations, which may influence both behavioral and biochemical parameters. However, increasing evidence now suggests that sex differences may affect antidepressant responses and neuroplasticity. Given this, future studies should include both male and female animals to determine if the observed lipid and nanomechanical alterations show sex-dependent patterns.

Further research is needed to determine whether the observed physicochemical changes in membranes occur gradually over time. If these changes are not visible after a short period (e.g., 7 days), this would strongly support the explanation for the delayed clinical onset of antidepressants, which typically occurs after several weeks. Such results would directly link the delayed clinical onset of SSRIs to a slow process of cell membrane remodeling. Continuing this line of thought, acute treatment with antidepressants had no effect in the AFM model (Supplementary Figure S3). This observation may suggest membrane remodeling following long-term treatment.

## 5 Conclusion

This study provides the first integrated evidence linking chronic SSRI treatment with lipid composition remodelling and membrane elasticity changes in brain regions critical for memory processing. By combining FT-IR spectroscopy and atomic force microscopy with behavioural memory testing, we demonstrate that fluoxetine and escitalopram exert distinct effects on cognitive performance that parallel region-specific alterations in neuronal membrane properties. These findings introduce lipid-dependent regulation of membrane physicochemistry as a previously underappreciated mechanism underlying SSRI-induced modulation of memory, opening new avenues for therapeutic strategies and biomarker development in depression and cognitive disorders.

## Data Availability

The original contributions presented in the study are included in the article/Supplementary Material, further inquiries can be directed to the corresponding author.

## Supplementary material

SUPPLEMENTARY FIGURE S1 (**A)** Dose dependence study of fluoxetine and escitalopram in C57Bl/6J mice using tail suspension test (TST). Values are expressed as the means ± S.E.M., and were evaluated by one-way ANOVA, ***P *<* 0.001, vs. vehicle group; (n = 6). **(B)** The effects of chronic treatment for 28 days with SSRIs on behavior of C57Bl/6J in TST – selected doses, fluoxetine 10 mg/kg; escitalopram 2 mg/kg *i.p*. Compounds were injected 30 min before the test and once daily (before 11:00 a.m.), for 28 days – when chronic treatment was scheduled. Values are expressed as the means ± S.E.M., and were evaluated by one-way ANOVA, *P *<* 0.05, ***P *<* 0.001 vs. vehicle group; (n = 8). (A) [F(4,26) = 24.09; P < 0,0001]; (B) [F(2,21) = 9.94; P = 0.0009]. Asterisk on Suppl. 1 represents Post Hoc Newman-Keuls test. Differences between acute and chronic effects of SSRIs are frequently reported in the literature. They may reflect adaptive neurobiological processes. Furthermore, indicates mechanisms such as receptor desensitization, altered serotonergic signaling, or compensatory plasticity. The lower behavioral effect after chronic escitalopram likely reflects adaptive responses rather than reduced activity.

SUPPLEMENTARY FIGURE S2 The effect of chronic treatment with Vehicle **(A)**, fluoxetine 10 mg/kg **(B)** or escitalopram 2 mg/kg **(C)** on body weight of C57Bl/6J mice. Values are expressed as the means ± S.E.M., and were evaluated by one-way ANOVA, ns – not significant; (n = 8).

SUPPLEMENTARY FIGURE S3 Elastic modulus of murine prefrontal cortex (pFCx – red histogram) and hippocampus (HP – green histogram): **(A,B)** bare tissue, **(C,D)** fluoxetine treatment and **(E,F)** escitalopram treatment. Acute treatment.
