# Supplementary figures and images for: Impact of chronic SSRI administration on memory parameters, lipid components and membrane properties in C57Bl/6J mice
Source: Front Pharmacol. 2026 Apr 17;17:1769754. doi: 10.3389/fphar.2026.1769754 (PMC13133016; doi:10.3389/fphar.2026.1769754)

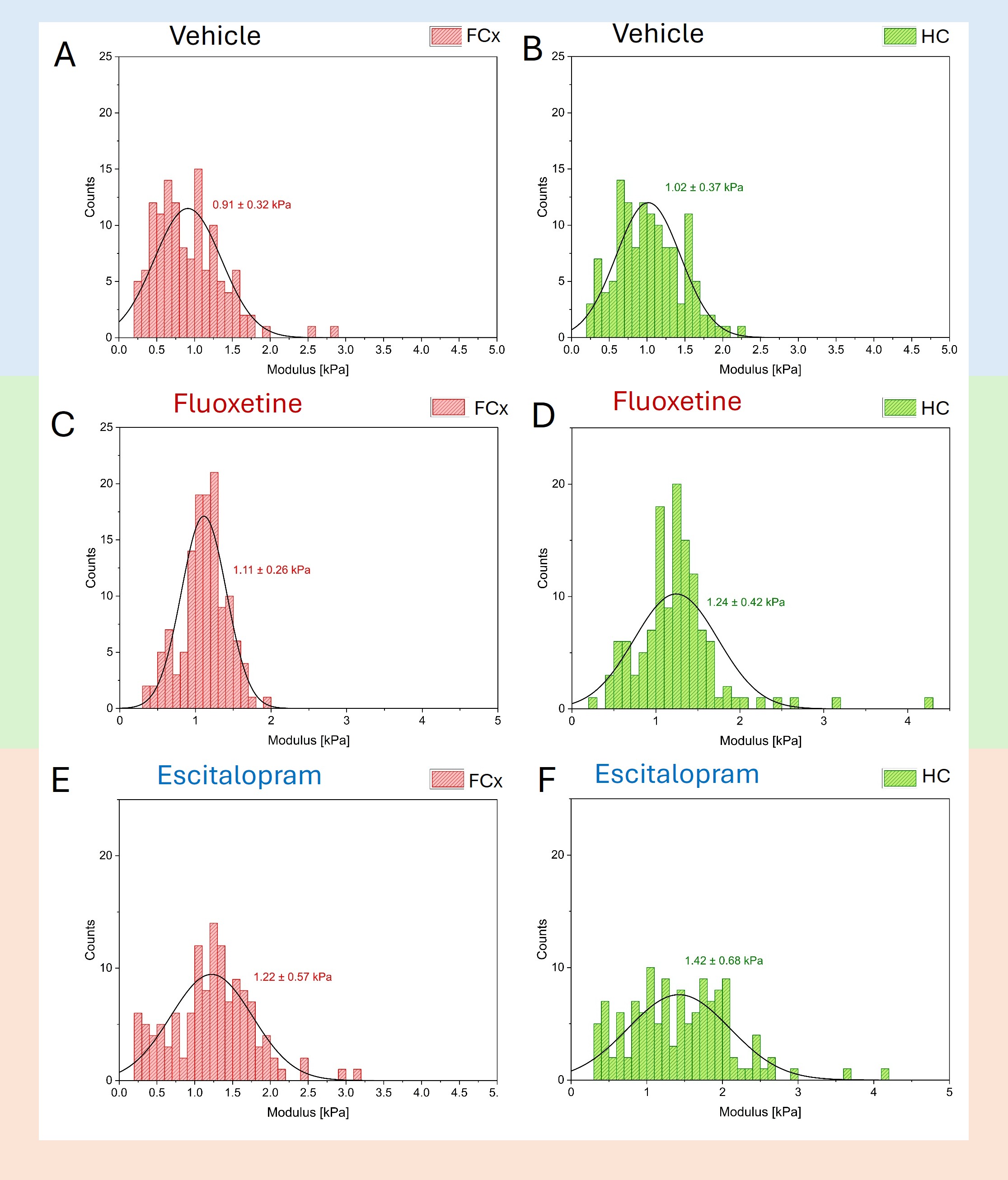

Supplement: Supplementary file 1 [file Image3.jpg]

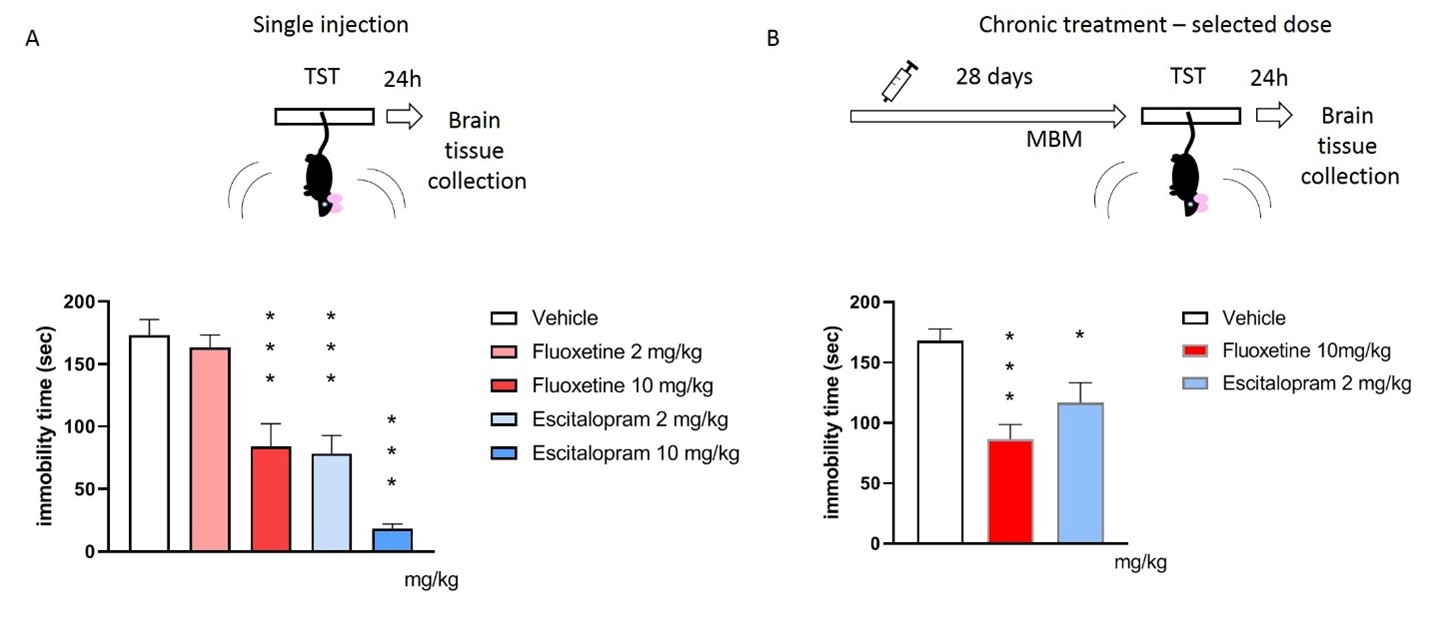

Supplement: Supplementary file 3 [file Image1.jpeg]

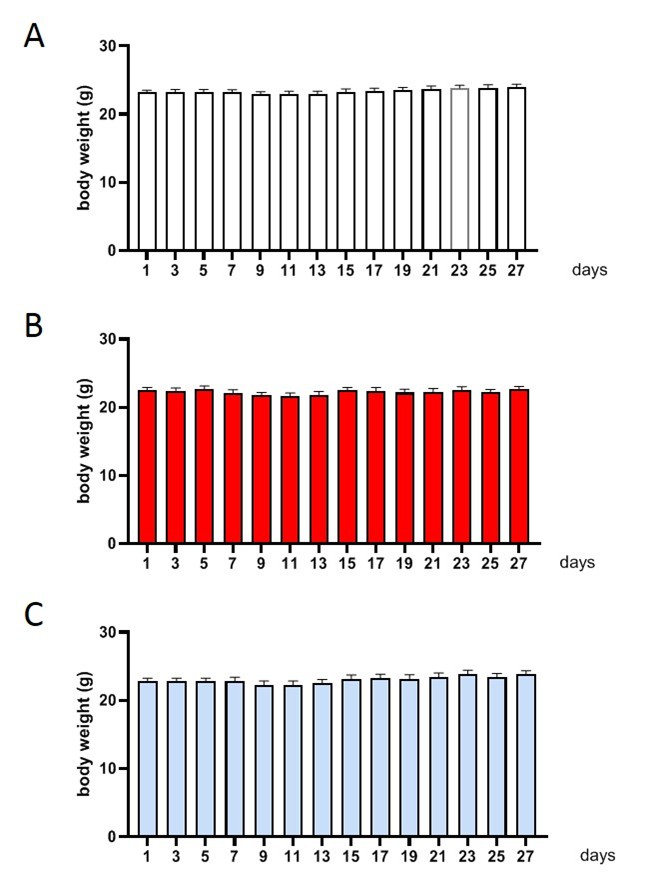

Supplement: Supplementary file 4 [file Image2.jpeg]
