# Supplementary material for: Impact of chronic SSRI administration on memory parameters, lipid components and membrane properties in C57Bl/6J mice
Source: Front Pharmacol. 2026 Apr 17;17:1769754. doi: 10.3389/fphar.2026.1769754 (PMC13133016; doi:10.3389/fphar.2026.1769754)
Supplement: Supplementary file 2 [file Table1.docx]

**Suppl. Table 1.** Effects of single (**A**) or chronic (**B**) injections with fluoxetine (10 mg/kg) or escitalopram (2 mg/kg) *i.p*. on locomotor activity (LA) of C57Bl/6J mice. Values are expressed as the means ± S.E.M., and were evaluated by one-way ANOVA, *P *<* 0.05, **P *<* 0.01 vs. vehicle group refer to post-hoc test. It was documented that a low doses of escitalopram in a stress may impact LA and in the same time the compound is an effective antidepressant (Beaver et al., 2022), that’s why above result may be ignored.

| Treatment | Dose (mg/kg) | Locomotor activity/6 min |
| --- | --- | --- |
| A | | |
| Vehicle | - | 3624±264.8 |
| Fluoxetine | 2 | 3571±315.4 |
| Fluoxetine | 10 | 4259±245.8 |
| Escitalopram | 2 | 5218±336.8** |
| Escitalopram | 10 | 5524±399.2** |
| P=0.0003 | | |
| B | | |
| Vehicle | - | 1827±78.30 |
| Fluoxetine | 10 | 1493±91.24* |
| Escitalopram | 2 | 2190±104.3* |
| P=0.0001 | | |
